# Complete genome sequence of turbot circovirus strain TurCV10LN-18/2021 from diseased *Scophthalmus maximus*

**DOI:** 10.1128/mra.00218-25

**Published:** 2025-09-26

**Authors:** Xiao Wu, Boyin Jiang, Sang Ho Choi, Yuanxing Zhang, Qiyao Wang, Yue Ma

**Affiliations:** 1 State Key Laboratory of Bioreactor Engineering, East China University of Science and Technology 47860, Shanghai, China; 2 National Research Laboratory of Molecular Microbiology and Toxicology, Seoul National University, Seoul, Republic of Korea; 3 Shanghai Engineering Research Center of Maricultured Animal Vaccines, Shanghai, China; 4 Laboratory of Aquatic Animal Diseases of MOA, Shanghai, China; 5 Laboratory for Marine Fisheries Science and Food Production Processes, Qingdao National Laboratory for Marine Science and Technology 474988 , Qingdao, China; Katholieke Universiteit Leuven, Leuven, Belgium

**Keywords:** circovirus, *Scophthalmus maximus*, viral pathogenesis

## Abstract

We reported the complete genome sequence of turbot circovirus (TurCV) strain TurCV10LN-18/2021, detected in a diseased turbot (*Scophthalmus maximus*) in China. TurCV is responsible for the emerging acute hemorrhagic syndrome (EAHS) in farmed turbot. The availability of the complete genome sequence facilitates the investigation of circovirus epidemiology in turbot.

## ANNOUNCEMENT

The viruses of the family *Circoviridae* are non-enveloped, single-stranded circular DNA viruses. They are the smallest known viruses infecting animals and plants, and the family includes two genera: *Gyrovirus* and *Circovirus* (1). Reports on circoviruses in fish are very limited. So far, complete circovirus genomes have only been detected in three fish species. In 2010, the fish circovirus sequence was discovered in barbel (*Barbus barbus*) in Hungary (2). Later, in 2012 and 2014, circoviruses were also reported in European catfish (*Silurus glanis*) and eel (*Anguilla anguilla*) (3, 4).

We report the complete genome sequence of turbot circovirus (TurCV) strain TurCV10LN-18/2021, detected in heart, spleen, and kidney tissue of multiple diseased turbots (*Scophthalmus maximus*) in an EAHS outbreak in Liaoning, China. In detail, 2 g of mixed tissue samples of heart, spleen, and kidney from the diseased turbots was homogenized vigorously with beads in 10 mL PBS for 20 min. The homogenates were centrifuged at 4°C and 8,000 revolutions per minute (RPM) for 20 min, then the supernatants containing infectious virus were collected as a crude purified virus suspension.

Complete genome sequencing was performed using strategies reported for other circoviruses (5–7). Genomic DNA was extracted from the 1 mL suspension using the TIANamp Genomic DNA extraction Kit (Tiangen Biotech, China) following the manual. Subsequently, nucleotide fragments were amplified by using the Pfu DNA polymerase (Tiangen Biotech, China). First-round PCR primers (Table 1) were designed targeting a 208 bp-conserved region revealed by multiple genome alignments of available circovirus (2–4), and further primer sets (Table 1) were designed for complete genome amplification and primer-walking Sanger sequencing. Overlapping sequence reads covering the entire genome were generated. Then, ~1 µg PCR product was run on agarose electrophoresis and purified with a QIAquick gel extraction kit (QIAGEN) and cloned using the pMD18-T vector (TaKaRa) according to the manufacturer’s instructions. Three positive clones for each fragment were sequenced by Sanger sequencer using M13 universal forward and reverse sequencing primers. Genomic reconstruction was done using DNASTAR software (version 5.0; DNASTAR Inc., Madison, WI). Default parameters were used except where otherwise noted.

**TABLE 1:** PCR primers used in the present work

Primers	Sequence (5′ to 3′)	Product size (bp)	Location (nt)
CapF	CCATGTCAAAGTTGACCTG	208	1,123–1,141
CapR	AGCAGAAGGGCGTGGATTG		1,312–1,330
Out-capF	CAATCCACGCCCTTCTGC	1,566	1,312–1,329
Out-capR	CAGGTCAACTTTGACATGG		1,123–1,141
0110-R2 = AP04 G0	ACGGGGACCCATTCACTAAG	989	1,044–1,063
0110-F2 = AP04 G01	ATCAACCATCCTGAATTTGAGG		75–96
0110-F1 = AP04 F11	GCCGCTTGTATAATCCACATGC	1,107	928–949
0110-R1 = AP04 F12	ATGTGCCCGGGGAGAGAACT		241–260
ORF1-F	ATGCCTAAATCAAGAGAAAACCCA	933	30–53
ORF1-R	TTATTGATCAACTGCATGTGGA		941–962
ORF2-F	ATGCAACTTCTCATTCTCTTTTAT	696	1,745–1,768
ORF2-R	CTAGGAAGCCAGCCCCA		1,073–1,089
ORF3-F	ATGCCTGATGAATCTTGTCTG	300	1,580–1,600
ORF3-R	CTAAGTAATCCTCAAATTCAGGA		83–105

The complete circular genome of this isolate is 1,774 nucleotides in length, with a G + C content of 49.0%. Three open reading frames (ORFs) were predicted from the nucleotide sequence with DNASTAR and by comparing the results with the genome organization and ORFs of other circoviruses. ORF1, stretching from 30 to 962 nt, encodes a classical replication-associated protein (Rep) of 310 amino acids (aa). ORF2 and ORF3 range from nt 1,073 to 1,768 and nt 1,580 to 105, encoding proteins of 231 and 99 aa, respectively.

**Fig 1:**
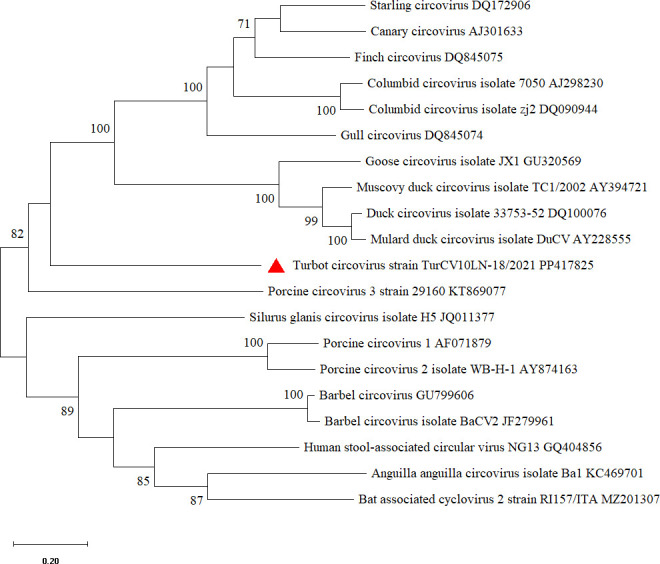
Phylogenetic tree of the turbot circovirus genome based on nucleotide level. Genomic sequences were first aligned by ClustalW (version 2.1). The evolutionary history was inferred by using the maximum likelihood method and Tamura-Nei model (8). The tree with the highest log likelihood (−37,142.06) is shown. The percentage of trees in which the associated taxa clustered together is shown above the branches. Initial tree(s) for the heuristic search were obtained automatically by applying Neighbor-Join and BioNJ algorithms to a matrix of pairwise distances estimated using the Tamura-Nei model, and then selecting the topology with superior log likelihood value. The tree is drawn to scale, with branch lengths measured in the number of substitutions per site. This analysis involved 20 nucleotide sequences. There was a total of 2,454 positions in the final data set. Evolutionary analyes were conducted with MEGA11 (9).

Representative strains of circoviruses, such as PCV1, PCV2, GoCV, and DuCV, were selected. The results of the phylogenetic analysis showed that TurCV is divergent and only distantly related to other known circoviruses, displaying nucleotide identities 67.52%–72.50% to DuCV strains, in the phylogenetic tree (Fig. 1).

The report of a complete TurCV genome sequence is critical for further investigation of its molecular characteristics and pathogenic potential.

## Data Availability

The whole-genome sequence of the turbot circovirus TurCV strain TurCV10LN-18/2021 has been deposited in DDBJ/ENA/GenBank under the accession number PP417825 (BioProject number PRJNA1099169). The sequencing reads have also been submitted to the Sequence Read Archive (SRA) under accession number SRR28624788.

## References

[1] Breitbart M, Delwart E, Rosario K, Segalés J, Varsani A, Ictv Report Consortium. 2017. ICTV virus taxonomy profile: Circoviridae. J Gen Virol 98:1997–1998. doi:10.1099/jgv.0.000871 28786778 PMC5656780

[2] Lőrincz M, Cságola A, Farkas SL, Székely C, Tuboly T. 2011. First detection and analysis of a fish circovirus. J Gen Virol 92:1817–1821. doi:10.1099/vir.0.031344-0 21525210

[3] Lőrincz M, Dán A, Láng M, Csaba G, Tóth AG, Székely C, Cságola A, Tuboly T. 2012. Novel circovirus in European catfish (Silurus glanis). Arch Virol 157:1173–1176. doi:10.1007/s00705-012-1291-1 22426897

[4] Doszpoly A, Tarján ZL, Glávits R, Müller T, Benkő M. 2014. Full genome sequence of a novel circo-like virus detected in an adult European eel Anguilla anguilla showing signs of cauliflower disease. Dis Aquat Organ 109:107–115. doi:10.3354/dao02730 24991738

[5] Li J, Xu S, Yuan X, Wang G, Shi J, Wu J, Cong X, Sun W, Du Y, Wang J. 2012. Complete genome sequence of recombinant porcine circovirus type 2 strain SD-3. J Virol 86:13870. doi:10.1128/JVI.02693-12 23166267 PMC3503058

[6] Wen L, He K, Ni Y, Zhang X, Li B, Wang X, Guo RL, Yu Z, Mao A, Zhou J, Lv L, Jiang J. 2012. Complete genome sequence of the rearranged porcine circovirus type 2. J Virol 86:5963. doi:10.1128/JVI.00494-12 22532529 PMC3347291

[7] Wang YY, Guo CJ, He JG. 2025. Aquatic circoviruses: emerging pathogens in global aquaculture-from discovery to disease management. J Virol 99:e0173724. doi:10.1128/jvi.01737-24 39670743 PMC11784310

[8] Tamura K, Nei M. 1993. Estimation of the number of nucleotide substitutions in the control region of mitochondrial DNA in humans and chimpanzees. Mol Biol Evol 10:512–526. doi:10.1093/oxfordjournals.molbev.a040023 8336541

[9] Tamura K, Stecher G, Kumar S. 2021. MEGA11: Molecular evolutionary genetics analysis version 11. Mol Biol Evol 38:3022–3027. doi:10.1093/molbev/msab120 33892491 PMC8233496

